# FeCl_3_-Modified Carbonaceous Catalysts from Orange Peel for Solvent-Free Alpha-Pinene Oxidation

**DOI:** 10.3390/ma14247729

**Published:** 2021-12-14

**Authors:** Adrianna Kamińska, Piotr Miądlicki, Karolina Kiełbasa, Jarosław Serafin, Joanna Sreńscek-Nazzal, Rafał Jan Wróbel, Agnieszka Wróblewska

**Affiliations:** 1 Department of Catalytic and Sorbent Materials Engineering, Faculty of Chemical Technology and Engineering, West Pomeranian University of Technology in Szczecin, Piastów Ave. 42, 71-065 Szczecin, Poland; kaminska.adrianna@zut.edu.pl (A.K.); piotr.miadlicki@zut.edu.pl (P.M.); Karolina.Kielbasa@zut.edu.pl (K.K.); rafal.wrobel@zut.edu.pl (R.J.W.); 2 Barcelona Research Center in Multiscale Science and Engineering, Department of Chemical Engineering, Institute of Energy Technologies, Technical University of Catalonia, Eduard Maristany 10-14, 08019 Barcelona, Spain; jaroslaw.serafin@upc.edu

**Keywords:** carbonaceous catalysts, iron compounds, oxidation of alpha-pinene, alpha-pinene oxide, verbenone, verbenol

## Abstract

The work presents the synthesis of FeCl_3_-modified carbonaceous catalysts obtained from waste orange peel and their application in the oxidation of alpha-pinene in solvent-free reaction conditions. The use of waste orange peel as presented here (not described in the literature) is an effective and cheap way of managing this valuable and renewable biomass. FeCl_3_-modified carbonaceous materials were obtained by a two-stage method: in the first stage, activated carbon was obtained, and in the second stage, it was modified by FeCl_3_ in the presence of H_3_PO_4_ (three different molar ratios of these two compounds were used in the studies). The obtained FeCl_3-_modified carbon materials were subjected to detailed instrumental studies using the methods FT-IR (Fourier-transform Infrared Spectroscopy), XRD (X-ray Diffraction), SEM (Scanning Electron Microscope), EDXRF (Energy Dispersive X-ray Fluorescence) and XPS (X-ray Photoelectron Spectroscopy), while the textural properties of these materials were also studied, such as the specific surface area and total pore volume. Catalytic tests with the three modified activated carbons showed that the catalyst obtained with the participation of 6 M of FeCl_3_ and 3 M aqueous solutions of H_3_PO_4_ was the most active in the oxidation of alpha-pinene. Further tests (influence of temperature, amount of catalyst, and reaction time) with this catalyst made it possible to determine the most favorable conditions for conducting oxidation on this type of catalyst, and allowed study of the kinetics of this process. The most favorable conditions for the process were: temperature of 100 °C, catalyst content of 0.5 wt% and reaction time 120 min (very mild process conditions). The conversion of the organic raw material obtained under these conditions was 40 mol%, and the selectivity of the transformation to alpha-pinene oxide reached the value of 35 mol%. In addition to the epoxy compound, other valuable products, such as verbenone and verbenol, were formed while carrying out the process.

## 1. Introduction

In recent years, there has been a growing interest in research on the use of industrial and agricultural waste to produce of activated carbons [1]. The availability and ease of the obtaining this waste makes it a good source of raw materials for the production of carbonaceous materials [2]. The main advantage of activated carbons obtained from biomass is their low production cost compared to commercial activated carbons. In addition, obtaining carbonaceous materials from biomass allows the utilization of raw materials that have not been used so far and is a new way to utilize biomass waste [3].

The Food and Agriculture Organization (FAO) estimates that the world’s citrus fruit production is close to 88 million tons per year, of which 80% is oranges [4]. The most important orange-producing countries are Brazil, USA and China. Brazil is the major citrus-processing country (Brazil processes 47% of the world’s citrus fruits). These fruits are mainly processed into juices. The other main food products, that are produced on a global scale are jams and marmalades [5]. The citrus fruit processing industry generates huge amounts of waste, with citrus peel accounting for as much as 60 to 65% [6]. Orange peel contains, among others, sugars, starch, cellulose, hemicellulose, lignin and pectin, which if inadequately stored may pollute the environment [7]. It is worth noting that the pH of orange peel is close to 4. Orange peel, which is generated as waste from the orange juice production process, must be properly disposed as it poses a threat to local water courses and leads to uncontrolled methane emissions through decomposition [8]. A solution to this problem may be the production of useful materials from orange peel that can be used in many areas of industry.

The synthesis of activated carbons from biomass on a laboratory scale, especially from orange peel, is very interesting for scientists working on obtaining new materials [9]. Activated carbons made of this raw material are characterized by a relatively well-developed specific surface area [10,11] and a large total pore volume [12], as well as the presence of micropores [13]. Moreover, the materials obtained contain very small proportions of other elements in their structure [14]. Activated carbons from waste orange peel have been successfully used in many sectors of electrical engineering [12,13,15,16], in chemical reactions [12,13,16] and as adsorbents in the adsorption of compounds from aqueous solutions [17]. Activated carbons can be also carriers of nanostructures in catalysts, which in turn can be used in chemical processes such as the adsorption of SO_2_ [18], electrochemical detection of toxic metal ions [19] or removal and recovery of cadmium from aqueous solutions [20].

The oxidation of alpha-pinene in the presence of catalysts is also of interest to many researchers. Alpha-pinene is obtained from the liquid resin (turpentine) of coniferous trees. The main source of alpha-pinene is the wood-processing industry, which produces waste with a high content of alpha-pinene [21]. Significant amounts of alpha-pinene are also present in the essential oils from plants [22,23,24,25]. The healing properties of alpha-pinene are well understood and described in the literature and alpha-pinene is used as a therapeutic substance in many diseases [26,27,28].

Alpha-pinene is a cheap raw material for the synthesis of many valuable compounds used as fragrances, food additives [29], pharmaceuticals [30] and solvents [7]. The oxidized derivatives formed in the oxidation reaction, such as verbenol, verbenone and alpha-pinene oxide, are of the greatest practical importance. These compounds are used primarily as flavor compounds and as the raw material for the production of fine chemicals (menthol, sandalol and taxol) [31,32,33]. Currently, the oxidation of alpha-pinene is carried out in the presence of various catalysts, including catalysts containing metals in their structure. The use of metal-containing catalysts and modification of the reaction conditions are aimed at obtaining the highest possible conversion and the highest selectivity transformation to alpha-pinene oxide. Table 1 shows selected catalysts for the alpha-pinene oxidation described in the literature and the parameters allowing to compare them in terms of activity in this reaction (selectivity to alpha-pinene oxide, alpha-pinene con-version), and the type of oxidant and solvent used.

The methods for the synthesis of FePO_4_ nanostructures have been described in the literature. These methods include: hydrothermal method [41], sol-gel method [42], surfactant-template method [43] and biologic template method [44]. Methods for the synthesis of FePO_4_ nanostructures using FeCl_3_ and H_3_PO_4_ have also been described. Wu et al. [45] obtained multi-wall carbon nanotubes supported by hydrated iron phosphate (FePO_4_). Wang [46] synthesized FePO_4_ microstructures (various crystalline forms). However, these methods require many reagents or are difficult to perform. The use of FePO_4_ nanoplates deposited on activated carbon as a catalyst for the oxidation of alpha-pinene has not been described in the literature so far. The presented method of synthesizing nanoparticles with simultaneous application to activated carbon, compared to the methods presented by other researchers, is simple to perform and requires the use of a small amount of chemical reagents.

The aim of this work was to obtain the active carbon catalysts from orange peels, which are bio-waste from the industry producing fruit juices. In order to increase the activity of the activated carbons obtained in the process of the carbonization of orange peels, their surface was modified by treating them with FeCl_3_ in the presence of H_3_PO_4_. The aim of the second stage of the research was to characterize the obtained modified carbon materials using the selected instrumental methods of DFT (Density-functional Theory), FT-IR (Fourier-transform Infrared Spectroscopy), XRD (X-ray Diffraction), SEM (Scanning Electron Microscope), EDXRF (Energy Dispersive X-ray Fluorescence) and XPS (X-ray Photoelectron Spectroscopy), while also the establishing the textural properties of these materials such as the specific surface area and total pore volume. The aim of the third stage was to conduct catalytic tests of the obtained modified carbon materials. The studies on catalytic activity focused on the process of alpha-pinene oxidation with oxygen. To our knowledge, catalysts based on activated carbons obtained from waste biomass from food processing, and modified with FeCl_3_ in the presence of H_3_PO_4,_ were not used to carry out this process. First, in the catalytic tests, it was necessary to select the catalyst sample with the highest activity, and then to conduct full catalytic tests with it (studies on the influence of temperature, amount of the catalyst and the reaction time), including also studies on the kinetics of the oxidation process. The aim of this step was to determine the most favorable conditions for alpha-pinene oxidation using this type of catalysts. In order to determine the most favorable conditions, the values for the conversion of alpha-pinene and the selectivity of transformation to alpha-pinene oxide were mainly taken into account.

## 2. Materials and Methods

### 2.1. Preparation Activated Carbon (AC)

The raw material used for the activated carbon (AC) production was orange peel (Valencia, Spain). A saturated solution of potassium hydroxide (Sigma-Aldrich, Burlington, MA, USA) was used for the chemical activation of this bio-waste. In our method of preparation fresh orange peel was dried in air, and then in an oven (Alpina, Konin, Poland) at 50 °C for 24 h. After drying, the orange peel was ground. 90 g of ground orange peel was mixed with 117 mL of KOH solution and subjected to an intensive mixing. The mass ratio calculated for dry biomass to KOH was 1:1. The obtained mixture was left for 3 h at room temperature.

The impregnated carbon substrate was dried at 200 °C for 19 h. After 19 h, the carbon substrate was subjected to carbonization under nitrogen gas (flow of 18 L/h). The carbon substrate was carbonized at 800 °C and kept at this temperature for 1 h. After the carbonization process was completed, the sample was cooled down to room temperature under an inert gas atmosphere. The activator (potassium hydroxide) was removed by washing of the sample with deionized water and a 1 M aqueous solution of HCl (Sigma-Aldrich, Burlington, MA, USA) for 19 h and after that again with deionized water until a neutral pH was reached. Washed AC was dried for 19 h at 200 °C. The obtained material was ground to powder. After the drying process was completed, the carbon sample was weighed and further analyzed. This material was identified as O_AC.

### 2.2. Preparation of Metallic Catalysts

First, 3 M, 6 M and 9 M aqueous solutions of FeCl_3_ (POL-AURA, Dywity, Poland) were added to 3 glass flasks. Each flask contained 1 g of the activated carbon obtained from orange peel. The flasks with these mixtures were placed on a magnetic stirrer (CHEMLAND, Stargard, Poland) and 20 mL of 3 M aqueous solution of H_3_PO_4_ (POL-AURA, Dywity, Poland) was added dropwise in variable molar ratios. For the 3 M solution of FeCl_3_ the molar ratio calculated for H_3_PO_4_ and FeCl_3_ was 1:1. Similarly, for the 6 M solution of FeCl_3_, the molar ratio was 1:2, and for the 9 M solution of FeCl_3_ it was 1:3. The flasks were tightly closed and heated in a stove at a constant temperature of 80 °C for 48 h. Next, the materials were rinsed several times with deionized water until pH of the filtrate became 7. In the next stage, the catalysts were dried in an oven at 100 °C for 24 h. These samples were identified as O_Fe3_H_3_PO_4_, O_Fe6_H_3_PO_4_ and O_Fe9_H_3_PO_4_.

### 2.3. Characterizing the Catalysts Obtained from Biomass

A Sorption Surface Area and Pore Size Analyzer (ASAP 2460, Micrometrics, Novcross, GA, USA, 2018) was used to characterize the textural properties of the obtained materials. Before the measurements of adsorption isotherms at the temperature of liquid nitrogen (−196 °C), all samples were degassed at 250 °C for 19 h. The Brunauer–Emmett–Teller (S_BET_) equation was used based on the obtained N_2_ adsorption-desorption isotherms to determine the specific surface area. The total pore volume (V_tot_) was determined by the volume of nitrogen adsorbed at a relative pressure of ~0.98. The DFT method based on nitrogen adsorption was used to calculate the volume of micropores. The pore size distribution was determined using the DFT model (ASAP 2460 software version 3.01, 2018, Micrometrics, Novcross, GA, USA) based on the N_2_ sorption isotherm. Applied DFT model: N_2_ at 77 K on carbon (slit N2-DFT Model adsorption).

Photographs were also taken with a scanning electron microscope (Neon40 Crossbeam, Carl Zeiss SMT GmbH, Oberchoken, Germany, 2009) in order to visualize the surface structures of the obtained materials.

Infrared spectra were acquired at room temperature with a Nicolet 380 ATR (Attenuated Total Reflectance)-FT-IR spectrometer (Thermo Fisher Scientific Inc., Waltham, MA, USA, 2003). Sixteen scans were averaged for each sample in the range 4000–400 cm^−1^.

X-ray diffraction (XRD) patterns of the catalyst were recorded by an X-ray diffractometer (X’Pert–PRO, Panalytical, Almelo, The Netherlands, 2012) using Cu K_α_ (λ = 0.154 nm) as the radiation source in the 2θ range 10–80°, with a step size of 0.026.

The composition of each catalyst was calculated using an energy-dispersive X-ray fluorescence (EDXRF) spectrometer (Panalytical, Almelo, The Netherlands, 2011).

The X-ray photoelectron spectroscopy measurements were performed in a commercial multipurpose (XPS, LEED (Low Energy Electron Diffraction), UPS (Ultraviolet Photoelectron Spectroscopy), AES (Auger Electron Spectroscopy)), UHV (Ultra High Vacuum) surface analysis system (PREVAC), which operates at a base pressure in the low 10^−10^ mbar range. The analysis chamber of the UHV system was equipped with nonmonochromatic X-ray photoelectron spectroscopy (XPS, PREVAC, Rogów, Poland, 2007) and kinetic electron energy analyzer (SES-2002, Scienta Scientific AB, Uppsala, Sweden, 2002). The calibration of the spectrometer was performed using Ag 3d5/2 transition. Samples in the form of fine powder were thoroughly degassed prior to measurement so that during XPS measurements the vacuum was in the low 10^−9^ mbar range. The X-ray photoelectron spectroscopy was performed using Mg K_α_ (hν = 1253.7 eV) radiation. Charging effects were observed and the correction of binding energy scale was performed using C 1s peak at 284.6 eV.

### 2.4. Alpha-Pinene Oxidation Method

The reaction of alpha-pinene oxidation was carried out in a three-necked flask placed in an oil bath with a bubbler and a reflux condenser (CHEMLAND, Stargard, Poland). Oxygen with a purity of 99.99% was fed from the cylinder through the mass flow meter, and the oxygen flow rate was 40 mL/min. For studies on oxidation, 8 g of alpha-pinene (98%, Sigma-Aldrich, Burlington, MA, USA) and the appropriate amount of catalyst were used. The activity of the catalysts was tested under the following conditions: reaction temperature 100 °C, catalyst amount 2.5 wt%, reaction time 3 h and mixing speed 400 rpm. The most active catalyst was used to determine the most favorable reaction conditions. For this purpose, the influence of the following parameters was studied: temperature in the range 80–120 °C, catalyst content in the range 0.1–2.5 wt% and reaction time from 20 to 280 min.

Quantitative analyses of the post-reaction mixtures were performed by the gas chromatography method with a Thermo Electron FOCUS chromatograph (FOCUS GC, Waltham, MA, USA, 2010) equipped with a FID (flame ionization detector) and a ZB-1701 column (30 m × 0.53 mm × 1 μm, 14% Cyanopropylphenyl, 86% Dimethylpolysiloxane). The operating parameters of the chromatograph were as follows: helium flow 1.2 mL/min, injector temperature 220 °C, detector temperature 250 °C, furnace temperature isothermally for 2 min at 50 °C, increase at a rate of 6 °C/min to 120 °C, then rising at 15 °C/min to 240 °C. The method of normalization was used for quantitative analyses of the post-reaction mixtures. Qualitative analyses were performed by GC-MS (Gas chromatography–mass spectrometry) method using a ThermoQuest apparatus (Waltham, MA, USA, 2000) equipped with a Voyager detector and a DB-5 column. The obtained results of the analysis were compared with the spectral libraries, and then the determined products were confirmed with the use of commercial standards.

## 3. Results and Discussion

### 3.1. Characterization of the Obtained Catalysts

**Figure 1:**
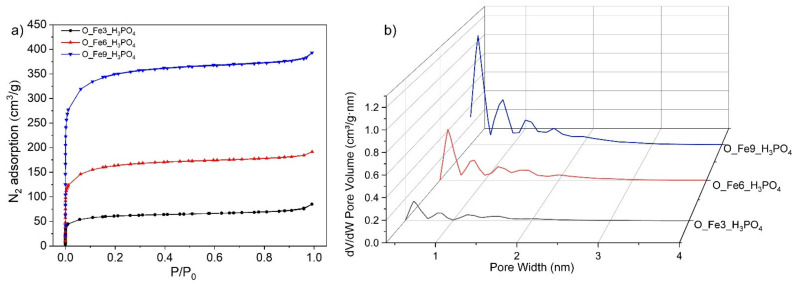
(**a**) The adsorption-desorption isotherms of N_2_ at −196 °C and (**b**) the pore volume distribution for the obtained modified carbonaceous catalysts.

The porous structure of the obtained catalysts was confirmed by the N_2_ adsorption-desorption measurements. Figure 1a presents nitrogen adsorption-desorption isotherms and Figure 1b shows the pore volume distribution according to their size in the range of micropores and narrow mesopores, for the obtained modified carbonaceous catalysts.

The isotherms of the tested materials demonstrated a high adsorption of N_2_ at low relative pressure that is characteristic for microporous materials. According to the IUPAC classification, the nitrogen sorption isotherms corresponded to type I(b). Type I(b) isotherms are characteristic for materials with pore distribution in the size of micropores and possibly narrow mesopores (<~2.5 nm) [47].

Curves presented in Figure 1b were determined by the analysis of adsorption isotherms N_2_ at −196 °C, using the non-linear method DFT (density functional theory). Based on these data, it was noticed that the analyzed catalysts, apart from smaller pores with a diameter of approx. 1–2 nm, also contained a small share of narrow mesopores with the size of ~2.5 nm.

Table 2 shows textural properties and metal content measured using XRF spectroscopy in modified carbonaceous catalysts obtained from biomass (waste orange peel).

Modified carbonaceous catalysts obtained from orange peel have surface area values in the range of 221–1300 m^2^/g and total pore volume values of 0.132–0.608 cm^3^/g. Sample O_Fe3_H_3_PO_4_ was characterized by the highest iron content in its structure (25.01 wt%), while the lowest iron content was recorded for the O_Fe9_H_3_PO_4_ sample (6.12 wt%).

Modifications in the use of iron precursors influenced the textural properties of the modified materials. A decrease in value of the specific surface area and total pore volume with the simultaneous increase of the content of Fe (wt%) in the material was also noted by Braun [48], Jiang [49] and Yuan [50].

**Figure 2:**
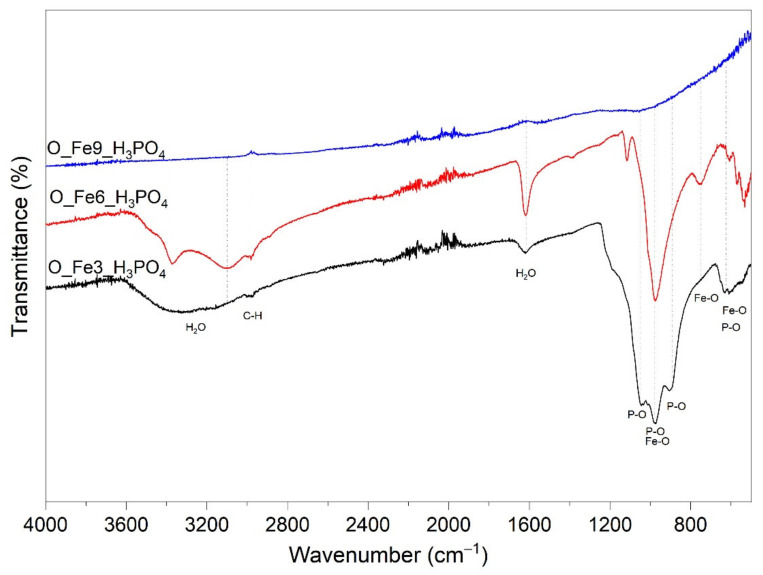
FT-IR spectra for the obtained modified carbonaceous catalysts.

The FT-IR spectrum of the obtained modified carbonaceous catalysts is shown in Figure 2. The characteristic band at 1628 cm^−1^ and wide double band between 2900 and 3750 cm^−1^ are attributed to the existence of adsorbed water. The internal vibrations of FePO_4_ originating from the intramolecular vibrations of PO_4_ tetrahedron are universally known to be located in the range of 400–1220 cm^−1^ [51,52]. Bands at 700, 980 and 1250 cm^−1^ are associated with C–P stretching vibrations. The absorption band between 2800 and 2900 cm^−1^ can be due to the aliphatic character of the C–H groups [53]. The bands below 600 cm^−1^ were related with the different Fe–O and P–O bending and stretching modes [54]. The bands around 1000 and 700 cm^−1^ can be assigned to the stretching mode of Fe–O [55]. To conclude on FT-IR characterization, the most important changes introduced by the increase of the acid concentration were the development of C–H vibrations (probably because of the loss of oxygen at the surface of the carbon material) as well as the increase of phosphoros group content (∼1100 cm^−1^). Benaddi et al. [56] suggested that dehydration of the biomass material by H_3_PO_4_ is similar to dehydration of alcohols and that at higher temperatures the phosphorous oxides act as Lewis acids and can form C–O–P bonds.

**Figure 3:**
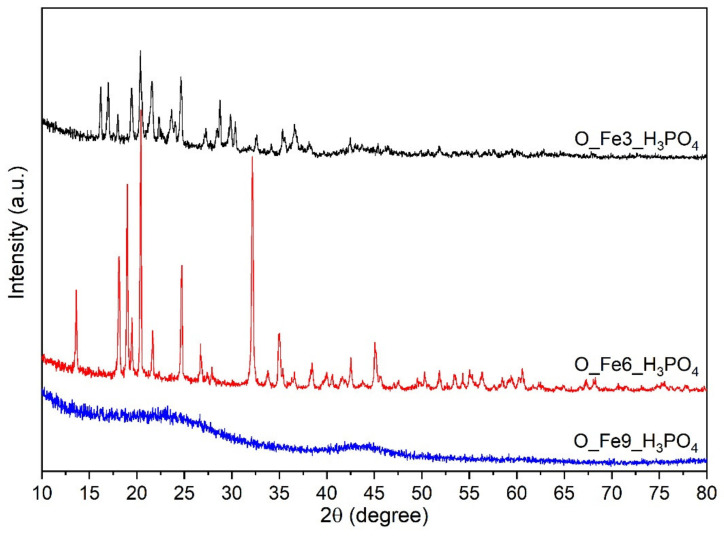
Diffractograms for the obtained modified carbonaceous catalysts.

Figure 3 shows the diffractograms of the obtained modified carbonaceous catalysts. The XRD plots of O_Fe6_H_3_PO_4_ and O_Fe3_H_3_PO_4_ showed characteristic peaks of iron phosphate hydrate. Although O_Fe3_H_3_PO_4_ and O_Fe6_H_3_PO_4_ samples contained FePO_4_·2H_2_O compound, the crystallographic systems were different. Namely these were orthorhombic (PDF4+ 04-014-3291) and monoclinic (PDF4+ 04-012-6194), respectively. The diffraction pattern of the O_Fe9_H_3_PO_4_ sample shows two peaks at positions 23° and 44° 2θ. They can be assigned to reflections from the corresponding graphite planes: (002) and (101) [57]. The material O_Fe9_H_3_PO_4_ has an amorphous structure, while materials O_Fe6_H_3_PO_4_ and O_Fe3_H_3_PO_4_ were crystalline. Similar results were presented by Wang [58] and Masquelier [59]. Well-developed crystals are confirmed by SEM micrographs.

**Figure 4:**
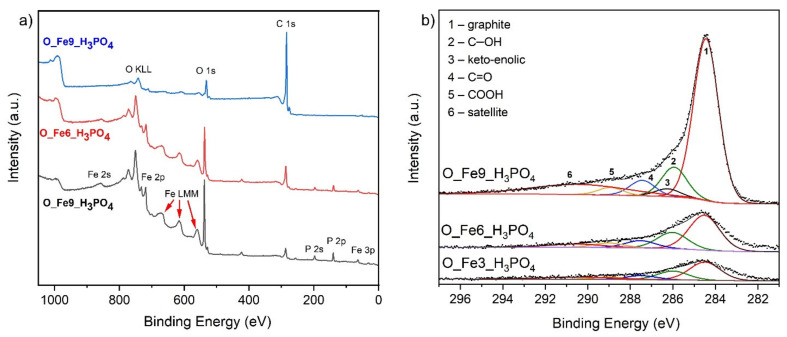
(**a**) X-ray photoelectron survey spectra of analyzed samples and (**b**) X-ray photoelectron spectroscopy C 1s signals with components.

The concentrations of different oxygen groups over the sample surfaces were determined using XPS. The results are presented in Table 3. These results were obtained by the careful deconvolution of C 1s signals presented in Figure 4a. The detailed deconvolution is presented elsewhere [60].

The oxygen atom, as an element more electronegative than carbon, causes a shift of valence electrons from carbon to oxygen. As a result, the electrons occupying 1s orbital exhibit increased binding energy. This effect is strongest for the COOH group, where two oxygen atoms participate in this phenomenon. Consequently, in Figure 4a, one can notice a dominant signal from elemental carbon and a shoulder located at higher binding energies corresponding to different carbon-oxygen groups.

The quantitative analysis of carbon-oxygen groups reveals that samples O_Fe3_H_3_PO_4_ and O_Fe6_H_3_PO_4_ were comparable in this respect. The sample O_Fe9_H_3_PO_4_ exhibited lower content of C–O; C=O; COOH groups and higher content of keto-enolic groups.

The intensity of the C 1s signals indicate a screening effect of the carbon surface for the O_Fe3_H_3_PO_4_ and O_Fe6_H_3_PO_4_ samples by iron phosphate species.

The X-ray photoelectron spectroscopy survey analysis (Figure 4b) enables determination of the elemental composition of the surface. The elemental content of the surface expressed as atomic concentration is presented in Table 4.

The sample O_Fe9_H_3_PO_4_ contains no phosphor and the lowest amount of iron. This explains the screening effect observed in case of the C 1s signal (Cf. Figure 4a). The significant amount of oxygen in the O_Fe6_H_3_PO_4_ and O_Fe9_H_3_PO_4_ samples is predominantly the impact of the presence of iron phosphate species. These species are located on the carbon surface, resulting in a screening effect. Consequently, the C 1s signal is the lowest for the sample containing the highest amount of phosphorus. One should be aware that the XPS signals originate from about 1 nm depth and that their contribution to the signal decreases exponentially with the depth. The Auger signals like Fe LMM are much more surface-sensitive; i.e., the signal originates from about 0.1 nm. One can observe strong iron Auger signals in case of the samples O_Fe3_H_3_PO_4_ and O_Fe6_H_3_PO_4_. This confirms that the iron phosphates are located over the carbon surface.

**Figure 5:**
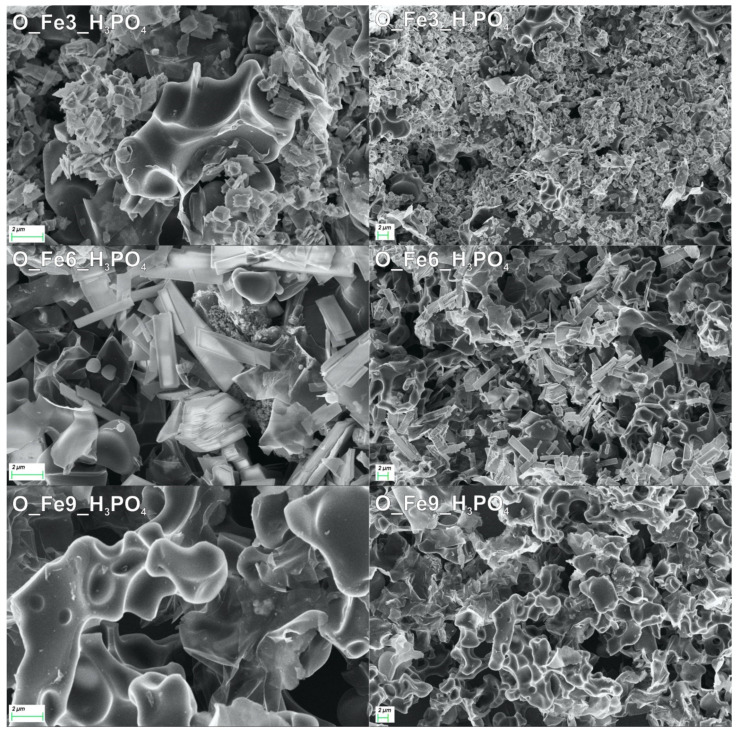
Scanning Electron Microscopy micrographs of the obtained modified carbonaceous catalysts.

Figure 5 shows SEM images of the obtained modified carbonaceous catalysts. The morphology of the catalysts’ surface elements was characterized by means of this micrograph. The SEM images of O_Fe9_H_3_PO_4_ show that the surface of this carbonaceous catalyst has cracks and crevices, and holes of different diameters. From the micrographs, it can be concluded that this material is characterized by a porous structure and the O_Fe9_H_3_PO_4_ catalyst has a typically carbonaceous structure. The SEM images of O_Fe6_H_3_PO_4_ show nanoplates present on the surface of the carbonaceous catalyst. These structures are characterized by a rectangular shape. The nanoplates can be defined as crystalline FePO_4_·2H_2_O. The micrograph of the O_Fe3_H_3_PO_4_ catalyst also shows structures of FePO_4_·2H_2_O. The structures on the surface of this catalyst showed an irregular shape resembling a square. Similar nanostructures were synthesized by Masquelier [59], Pramanik [61] and Wang [58].

The careful reader might notice that a higher concentration of Fe(III) during the preparation process leads to a lower iron content detected by XRF and XPS methods. The most likely explanation for this phenomenon is that the Fe(III) concentration strongly affects the crystallization process. The XRD data indicates (Figure 3) orthorhombic and monoclinic crystallographic systems for the FePO_4_·2H_2_O compound, and for O_Fe3_H_3_PO_4_ and O_Fe6_H_3_PO_4_ samples, respectively. However, the sample O_Fe9_H_3_PO_4_ obtained in the highest concentration of Fe(III) gives no XRD signals originating from FePO_4_·2H_2_O compound. The XRF data (Table 2) indicates a significant amount of iron which should be easily detected by the XRD method in the case of coarse grained material. Therefore, one can conclude that in case of sample O_Fe9_H_3_PO_4,_ the material was amorphous and was characterised with very fine crystallites or particles. The amorphous material obtained during the process of preparation can be easily washed out. This explains the decrease in iron content in the sample with increasing Fe(III) concentration during preparation our samples. The SEM data (Figure 5) supports this observation.

### 3.2. Activity of the Obtained Modified Carbonaceous Catalysts

**Figure 6:**
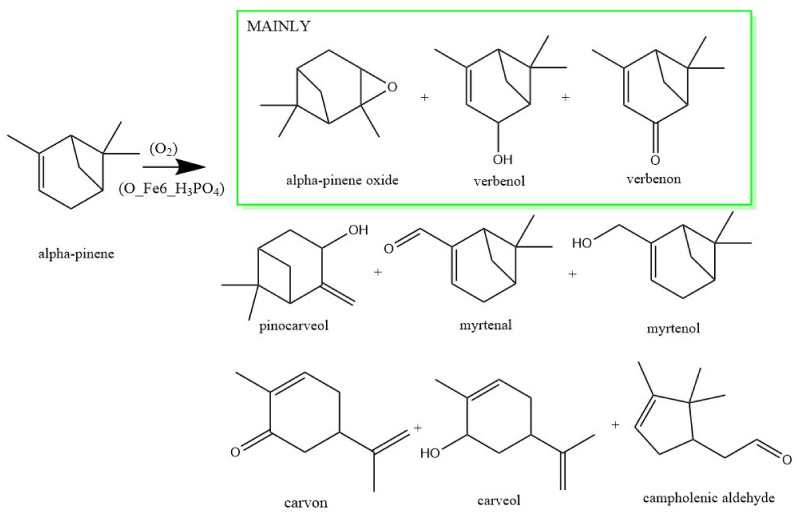
Main products of alpha-pinene oxidation with studied catalysts.

**Figure 7:**
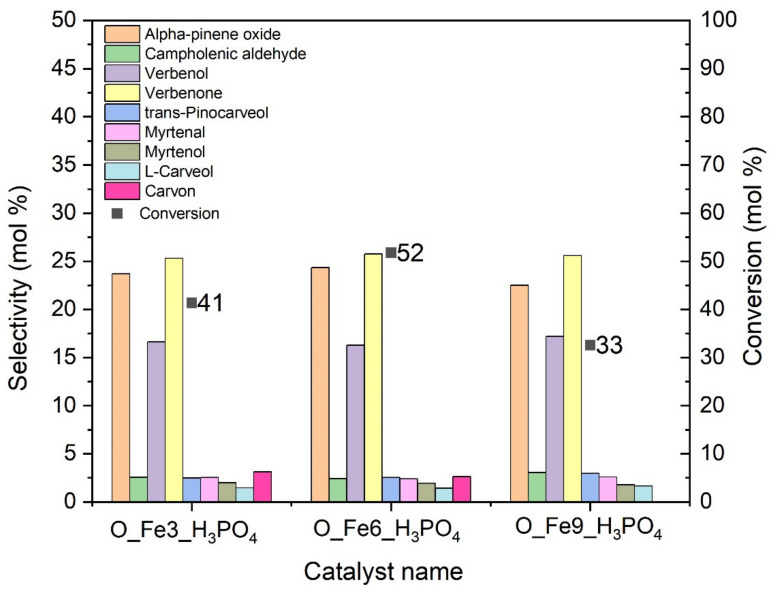
Comparison of selectivities of main products and alpha-pinene conversion after 3 h for the modified carbonaceous catalysts.

In the first stage of the research, the activity of the obtained catalysts in the oxidation of alpha-pinene was checked. The activity of the catalysts was tested under the following conditions: reaction temperature—100 °C, catalyst amount—2.5 wt%, reaction time—3 h, mixing speed—400 rpm. Figure 6 shows main products of alpha pinene oxidation. The compounds obtained with the highest selectivity are marked in the green box. Figure 7 shows a comparison of the selectivities for the main products and the conversion of alpha-pinene for tested catalysts.

The O_Fe6_H_3_PO_4_ catalyst was characterized by the highest catalytic activity at this stage of the study, because the highest values of conversion of alpha-pinene (52 mol%) and selectivity to alpha-pinene oxide as the one of main reaction products (24 mol%) were achieved on this catalyst. Other products with high selectivity in the reaction carried out with the use of O_Fe6_H_3_PO_4_ catalyst were: verbenone (25 mol%) and verbenol (16 mol%).

With the use of other catalysts, the selectivity values for alpha-pinene oxide were similar to the selectivity value obtained for the O_Fe6_H_3_PO_4_ catalyst and amounted to, respectively: 23 mol% for the O_Fe3_H_3_PO_4_ catalyst and 22 mol% for the O_Fe9_H_3_PO_4_ catalyst.

For O_Fe3_H_3_PO_4_ and O_Fe9_H_3_PO_4_ catalysts, lower conversion values (respectively 41 mol% and 33 mol%) were obtained compared to the conversion value obtained for the O_Fe6_H_3_PO_4_ catalyst. These differences are probably related to the presence of different functional groups on the catalyst surface and the different iron content in the catalyst support. However, looking at the results in Table 2 and Table 3, the difference in the content of functional groups on the catalyst surface seems to be more important here. Comparing the content of C–O, C=O and COOH groups in three modified carbon catalysts, it can be said that O_Fe3_H_3_PO_4_ and O_Fe6_H_3_PO_4_ catalysts have the highest content of these groups, while the O_Fe6_H_3_PO_4_ catalyst is characterized by their slightly higher content than in the O_Fe3_H_3_PO_4_. The C–O, C=O and COOH groups can form peroxy groups during the oxidation process, thanks to which we observe the occurrence of the appropriate oxidation reactions in the tested process. Therefore, the O_Fe9_H_3_PO_4_ catalyst should be the least active in the studied process of alpha-pinene oxidation as is confirmed by the results presented in Figure 7.

**Figure 8:**
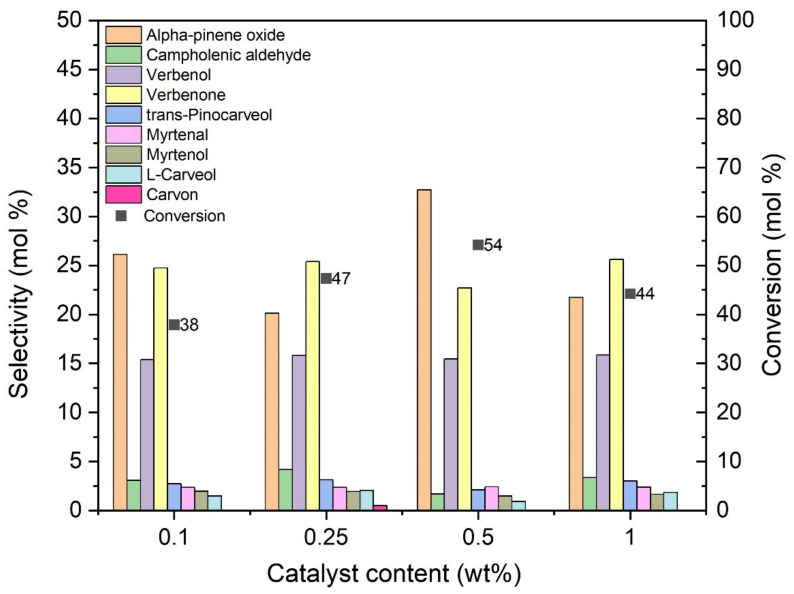
Influence of the O_Fe6_H_3_PO_4_ catalyst content on the selectivity of the main products for alpha-pinene conversion after 3 h.

The second stage of catalytic research focused on determining the most favorable conditions for alpha-pinene oxidation process using the most active catalyst. Taking into account the results obtained in the preliminary studies on the catalytic activity all three modified carbonaceous catalysts, the O_Fe6_H_3_PO_4_ catalyst was selected for the tests. The following parameters were tested: catalyst content in the range 0.1–1 wt%, temperature in the range 80–120 °C and reaction time from 20 to 280 min. The first parameter tested was the catalyst content in relation to the amount of alpha-pinene. The reaction was carried out at the temperature of 100 °C, and samples were taken after 3 h. The obtained results are shown in Figure 8.

Figure 8 shows that increasing the catalyst content in the range of 0.1 wt% to 1 wt% increases alpha-pinene conversion, which reaches a maximum value (54 mol%) for the catalyst content of 0.5 wt%. The highest selectivity of transformation to alpha-pinene oxide (33 mol%) was also noted for this catalyst content. For the catalyst content of 1 wt%, the values of alpha-pinene conversion (44 mol%) and selectivity to alpha-pinene oxide (22 mol%) decreased significantly. At this stage of the research, the amount of catalyst equal to 0.5 wt% was considered to be the most advantageous.

**Figure 9:**
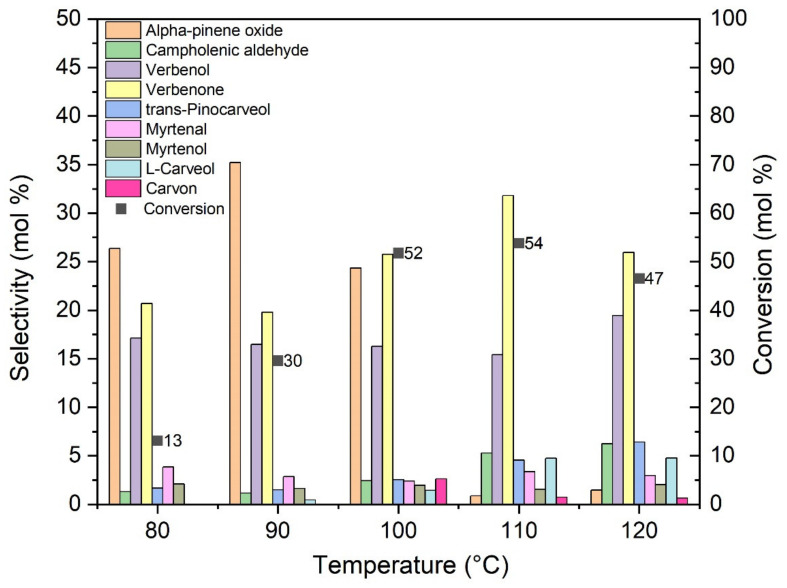
Comparison of the selectivity of the main products for the conversion of alpha-pinene at different temperatures after 3 h, using the O_Fe6_H_3_PO_4_ catalyst.

Figure 9 shows the influence of temperature on the oxidation of alpha-pinene in the presence of the O_Fe6_H_3_PO_4_ catalyst.

The oxidation of alpha-pinene was carried out for 3 h in the presence of 0.5 wt% catalyst. With increasing temperature, the conversion of alpha-pinene increases, reaching the maximum value of 54 mol% for the temperature of 110 °C, while at the temperature of 120 °C it decreases to 47 mol%. Similar values of alpha-pinene conversion (52 mol%) were also recorded for the temperature of 100 °C. The selectivity of transformation to alpha-pinene oxide is the highest at 90 °C (35 mol%), while for oxidation carried out at temperatures above 90 °C, the value of this selectivity decreases. This may be due to the transformation of alpha-pinene oxide to derivative compounds and compounds with higher molecular weights (dimers and polymers). Considering mainly the alpha-pinene conversion values, at this stage of the research, the temperature of 100 °C was taken as the most favorable.

**Figure 10:**
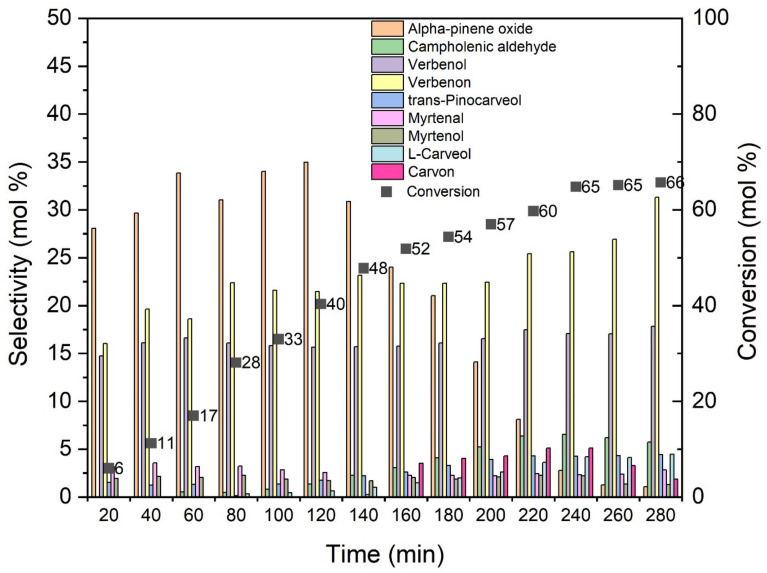
Influence of time on the selectivity of the main products for alpha-pinene conversion, using O_Fe6_H_3_PO_4_ catalyst.

Figure 10 shows the influence of the reaction time on the oxidation of alpha-pinene. For the studies on the influence of reaction time, 8 g of alpha-pinene and 0.04 g of catalyst (0.5 wt%) were used and temperature of oxidation was 100 °C. Samples of the reaction mixtures for GC studies were taken in the range 20–280 min at 20 min intervals.

The maximum value of selectivity to alpha-pinene oxide (35 mol%) was reached after 120 min of the oxidation process. After this time, the selectivity of the transformation to epoxide decreases and after 140 min this value amounts to 31 mol%, and after 280 min to only 1 mol%. The selectivity to verbenone increases with the prolongation of the reaction time and reaches its maximum value (31 mol%) for the reaction time of 280 min. The conversion of alpha-pinene also increases with the prolongation of the reaction time, reaching the maximum value (66 mol%) for a reaction time of 280 min. An increase in the alpha-pinene conversion values with a simultaneous decrease in the selectivity of alpha-pinene oxide values indicates further reactions in which alpha-pinene oxide undergoes isomerization, dimerization and polymerization.

### 3.3. Determination of the Kinetics Parameters

The comprehensive kinetic modeling of the alpha-pinene oxidation over FeCl_3_-modified carbonaceous catalysts obtained from orange peel was performed based on the series of experiments in which the effect of the temperature was checked, considering a constant oxygen uptake (expressed in mol/L). For each experiment (80, 90 and 100 °C; P_O2_ = 1 bar), the reaction mixture composition was determined at the varied points of oxygen uptake/alpha-pinene ratio (defined in mol_O2_/mol_α-pinene_). The alpha-pinene oxidation rates were calculated by kinetic curves differentiation. The turbulence created around the catalyst particles by a vigorous stirring of the reaction mixture helps to eliminate the external diffusion resistance between the bulk liquid and surface of the catalyst. Internal diffusion resistance was also negligible because of the small size of catalyst particles (0.07 mm ≤ d_p_ ≤ 0.1mm) used in the runs. It was observed that product content depends slightly on the oxygen uptake/alpha-pinene ratio. Moreover, the increase of the reaction temperature results in the growth of the alpha-pinene oxidation rate. Activation energy estimated from Arrhenius dependence was 92.7 ± 3.4 kJ/mol and the effective kinetic constant was k_eff_ = 1.0 × 10^10^ mol^0.5^·L^−0.5^·min^−1^. The model fits the experimental data quite well (the regression coefficient equals 0.9819); thus, the activation energy calculated is the true activation energy. Therefore, under the reaction conditions the reaction rate of alpha-pinene oxidation by molecular oxygen can be expressed as follows:

RO = k_eff_ × f(concentration)^0.5^ × P^0^_O2_ × exp(−E_a_/RT) (mol·L^−1^·min^−1^), (1)

Calculated activation energy matches results achieved for typical values of activation energy for alpha-pinene oxidation by molecular oxygen (81.3 kJ/mol) [62] as well as cis-pinene oxidation (79.5 kJ/mol) [63] or dibenzyl ester oxidation initiated by azoisobutyronitrile (93.66 kJ/mol) [64].

## 4. Conclusions

Summarizing our research presented in this work, it can be seen that we were able to obtain active catalysts for alpha-pinene oxidation with oxygen. Among the tested catalysts, the O_Fe6_H_3_PO_4_ catalyst was the most active, because the highest values of conversion of alpha-pinene (52 mol%) and selectivity transformation to alpha-pinene oxide as the one of main reaction products (24 mol%) were achieved on this catalyst. Other products with high selectivity in the reaction carried out with the use of O_Fe6_H_3_PO_4_ catalyst were verbenone (25 mol%) and verbenol (16 mol%). The studies on the influence of the amount of this catalyst on the course of the oxidation process showed that amount of the catalyst is the important parameter for this process. It was seen that increasing the catalyst content in the range of 0.1 wt% to 1 wt% increased the alpha-pinene conversion (to the maximum value of 54 mol% for the catalyst content of 0.5 wt%). The highest selectivity of transformation to alpha-pinene oxide (33 mol%) was also noted for this catalyst content. Temperature as the process parameter also had considerable influence on the process. With increasing temperature, the conversion of alpha-pinene increased, reaching a maximum value of 54 mol% at the temperature of 110 °C. The selectivity of transformation to alpha-pinene oxide was highest at 90 °C (35 mol%), while for oxidation carried out at temperatures above 90 °C, the selectivity decreased and the main product was verbenone. The last parameter studied was reaction time. This parameter was very important for the course of oxidation of alpha-pinene. The maximum value for the selectivity of transformation to alpha-pinene oxide (35 mol%) was reached at 120 min. After 160 min, the main product was verbenone. The selectivity of transformation to verbenone increased with the prolongation of the reaction time and reached its maximum value (31 mol%) at the reaction time of 280 min. The conversion of alpha-pinene also increased with the prolongation of the reaction time, reaching its maximum value (66 mol%) at 280 min. The increase in alpha-pinene conversion with the simultaneous decrease in the selectivity of alpha-pinene oxide indicated further reactions of alpha-pinene oxide (isomerization, dimerization, and polymerization).

The studies presented in this paper show that waste biomass in the form of fresh orange peel can be an excellent raw material for obtaining catalysts active in the oxidation of alpha-pinene. Catalysts obtained from orange pulp and modified with FeCl_3_ may become an alternative to synthetic catalysts used in this process in the future. This direction of technology development for the olefin oxidation process will ensure effective management of waste biomass and will allow the use of relatively cheap catalysts based on raw materials of natural origin in these processes. The manner of carrying out this process of alpha-pinene oxidation also needs to be emphasized, as it does not use any solvents, which reduces environmental nuisance and lowers the costs associated with the recovery of the solvent and its recycling. Alpha-pinene, which is oxidized in this process, is likewise obtained from raw materials of natural origin, which makes it a renewable resource. The oxidation process uses oxygen as the oxidizing agent, supplied from the cylinder and, under the most favorable conditions, the reaction is carried out at a temperature of 100 °C, at atmospheric pressure, with a catalyst content of 0.5 wt% and for 120 min. These are mild process conditions. The conversion of the organic raw material obtained under these conditions is 40 mol%, and the selectivity of the transformation to alpha-pinene oxide reaches the value of 35 mol%. In addition to the epoxy compound, other valuable products, such as verbenone and verbenol, can also be obtained in this process. Therefore, research on this process should be continued. This research should move towards modifications of carbon catalysts to be able to obtain higher selectivity of the main product or one of the most valuable by-products.

## Figures and Tables

**Table 1:** Selected catalysts for alpha-pinene oxidation.

Catalyst	Main Products	Selectivity to Alpha-Pinene Oxide (mol%)	Conversion of Alpha-Pinene (mol%)	Solvent/Oxidant	Ref.
V-MCM-41 *	Verbenone, trans-Sobrerol, Campholenic aldehyde	5	13	Acetonitrile/H_2_O_2_	[34]
Ti-MCM-41 *	Verbenone, Verbenol, Campholenic aldehyde	27	39	Acetonitrile/H_2_O_2_	[35]
MCM-41 * and HMS ** containing metal ions	Alpha-pinene oxide, 1,2-pinane diol	100	11	Chloroform/ TBHP *** or H_2_O_2_	[36]
Ti-HMS **	Verbenone, Verbenol, Campholenic aldehyde	13	30	Acetonitrile/ TBHP ***	[37]
FePcCl_16_-NH_2_-SiO_2_	Verbenone	16	61	Acetone/TBHP ***	[38]
H_5_PW_11_TiO_40_/silica	Verbenone, Verbenol	-	60	Acetonitrile/H_2_O_2_	[39]
Co-Ag supported ZnO	Verbenone, Myrtenal	-	100	Acetonitrile/H_2_O_2_	[40]
FeCl_3_-modified carbonaceous catalysts	Alpha-pinene oxide, Verbenone, Verbenol	35	40	Absent/O_2_	In this work

* Mobil composition of matter No. 41; ** Hexagonal mesoporous silica; *** *tert*-Butyl hydroperoxide.

**Table 2:** Textural properties and Fe content in the obtained modified carbonaceous catalysts.

Sample	S_BET_ (m^2^/g)	V_tot_ (cm^3^/g)	Fe (wt%)
O_Fe3_H_3_PO_4_	221	0.132	25.01
O_Fe6_H_3_PO_4_	602	0.296	17.94
O_Fe9_H_3_PO_4_	1300	0.608	6.12

**Table 3:** The content of C 1s components expressed as atomic concentrations.

Assignment	O_Fe3_H_3_PO_4_	O_Fe6_H_3_PO_4_	O_Fe9_H_3_PO_4_
C	46.7	50.8	61.9
C–O	22.4	23.7	11.4
Keto-enolic	0.0	0.0	3.0
C=O	10.2	10.6	5.9
COOH	6.0	6.6	2.9
Satellite	14.7	8.3	14.9

**Table 4:** The elemental content of the surface expressed as atomic concentration.

Sample	At. %			
	C	O	Fe	P
O_Fe3_H_3_O_4_	28.8	45.6	13.1	12.5
O_Fe6_ H_3_O_4_	53.2	30.9	8.4	7.6
O_Fe9_H_3_PO_4_	89.8	9.2	1.0	0.0

## Data Availability

The data presented in this study are available on request from the corresponding author.
